# Rapid and efficient testing of the toxicity of graphene-related materials in primary human lung cells

**DOI:** 10.1038/s41598-022-11840-2

**Published:** 2022-05-10

**Authors:** Javier Frontiñan-Rubio, Viviana Jehová González, Ester Vázquez, Mario Durán-Prado

**Affiliations:** 1 Faculty of Medicine, Universidad de Castilla-La Mancha, 13071 Ciudad Real, Spain; 2 Instituto Regional de Investigación Científica Aplicada (IRICA), Universidad de Castilla-La Mancha, 13071 Ciudad Real, Spain; 3 Faculty of Chemical Science and Technology, Universidad de Castilla-La Mancha, 13071 Ciudad Real, Spain

**Keywords:** Cell death, Respiratory tract diseases

## Abstract

Graphene and its derivative materials are manufactured by numerous companies and research laboratories, during which processes they can come into contact with their handlers' physiological barriers—for instance, their respiratory system. Despite their potential toxicity, these materials have even been used in face masks to prevent COVID-19 transmission. The increasingly widespread use of these materials requires the design and implementation of appropriate, versatile, and accurate toxicological screening methods to guarantee their safety. Murine models are adequate, though limited when exploring different doses and lengths of exposure—as this increases the number of animals required, contrary to the Three R's principle in animal experimentation. This article proposes an in vitro model using primary, non-transformed normal human bronchial epithelial (NHBE) cells as an alternative to the most widely used model to date, the human lung tumor cell line A549. The model has been tested with three graphene derivatives—graphene oxide (GO), few-layer graphene (FLG), and small FLG (sFLG). We observed a cytotoxic effect (necrosis and apoptosis) at early (6- and 24-h) exposures, which intensified after seven days of contact between cells and the graphene-related materials (GRMs)—with cell death reaching 90% after a 5 µg/mL dose. A549 cells are more resistant to necrosis and apoptosis, yielding values less than half of NHBE cells at low concentrations of GRMs (between 0.05 and 5 µg/mL). Indeed, GRM-induced cell death in NHBE cells is comparable to that induced by toxic compounds such as diesel exhaust particles on the same cell line. We propose NHBE as a suitable model to test GRM-induced toxicity, allowing refinement of the dose concentrations and exposure timings for better-designed in vivo mouse assays.

## Introduction

Although it was initially assumed that the primary interaction of graphene and graphene-related materials (GRMs) with humans was limited to their production and handling^1,2^, there is an increasing number of applications of these compounds in skin sensors, clothes, and accessories. This makes it necessary to establish safe-by-design production protocols and explore the interaction of this family of materials with different human physiological barriers prior to their commercialization^3–8^. One recent example of their commercial application is graphene-coated face masks to prevent the transmission of COVID-19—which were withdrawn in some countries because of their possible toxic effect on the respiratory tract^9–11^.

Extensive research has been carried out on this topic, particularly on the interaction of graphene-related materials (GRMs) with the lung barrier. However, this has yielded contradictory results. First, because there are multiple types of GRMs—with varying sizes, oxidation degrees, or number of layers, among other aspects, which interact with cells in different ways^3^. Second, an even more significant problem is the lack of standardization in toxicological screening methods, making it difficult to compare the effects of different GRMs. As a result, choosing the best material for commercial applications such as healthcare products, e.g., face masks, is often tricky. A standard model needs to be established for a method to become standardized. There are currently 141 articles available in PubMed which analyze the interaction of graphene and GRMs with the lung (search keywords: graphene, lung, and toxic), 82 of which evaluated graphene-induced toxicity in the lung in vivo or in vitro. Of these 82 articles, 24 used mouse (nine of these examining graphene exposure through the respiratory tract) and 12 used rat (six of these examining graphene exposures through the respiratory tract) in vivo models; one publication used 3D in vitro airway models^12^; and 45 of these publications used cultured in vitro lung cells—with diverse concentrations of GRMs that were added in acute (41 publications) or sub-acute (4 publications) doses.

The in vitro model—culturing lung cells in monolayers—is the most simple, reproducible, and versatile model. The major advantage of this approach is that it makes it easy to assay multiple concentrations and timings, from acute (high dose, short periods) to chronic (low dose, long periods). However, the main problem is that the gold standard for this model, the human tumor cell line A549, does not have the same physiology as normal airway lung cells. Indeed, A549 cells are highly resistant to the effect of compounds such as GO, even though it can be internalized^13–19^. In vivo models, either with mice or rats, are probably more appropriate. However, their major limitation is that a large number of animals are necessary to explore different concentrations and exposure times, which opposes the Three R's principle in animal experimentation^20^. Indeed, to perform in vivo experiments, it is mandatory to obtain first strong enough in vitro results to set up an animal protocol and obtain the Ethical Committees' approval.

Moreover, making animals inhale the desired amount of GRMs poses an additional problem. 3D in vitro airway models are a promising intermediate between in vitro and in vivo models. To our knowledge, only one paper to date has implemented this approach, using adenovirus-12 transformed cells (BEAS-2B) sprayed with an aerosol exposure system. However, this method is expensive and time-consuming, as it only allows testing one condition per experiment^12^.

This article presents an easy, reproducible, and versatile in vitro 2D model and a battery of contrasted cellular assays that could serve as the basis to establish a new standard to compare all GRMs—those already known to date and new ones that could be generated in the future. Primary human lung epithelial cells are more complex to culture than cancer A549 or adenovirus transformed BEAS-2B cells. However, they are still more manageable to set up than 3D models cultured with aerosol exposure systems. Normal human bronchial epithelial (NHBE) cells are primary, non-immortalized lung epithelial cells that behave as normal lung cells^21^. Previous research has used these as a model for testing drug delivery and absorption barrier^22^ and toxicity^23–27^. Our study examined the toxicity of several GRMs using different concentrations and exposure lengths on both NHBE and A549 lung tumor cells. Specifically, we used one commercial graphene oxide (GO), a few-layer graphene (FLG), and a small FLG (sFLG) synthesized both in our labs^28,29^, all of which had different oxidation degrees (GO >  > sFLG**≈**FLG) and lateral sizes (GO > FLG > sFLG). Our results indicate that NHBE cells were susceptible to all GRMs assayed, reaching an exacerbated mortality after seven days of incubation which was also significant at short incubation times (6 h). GRM dose and exposure length were the same, NHBE cell mortality was consistently higher, almost double, than A549 cells. These results highlight the need to use appropriate models to assay GRM-induced toxicity and provide easy-to-manage tools and protocols to conduct comparative studies among the growing number of emergent GRMs—prior to their testing in more complex in vivo models.

## Results

### Characterization of nanomaterials

**Figure 1:**
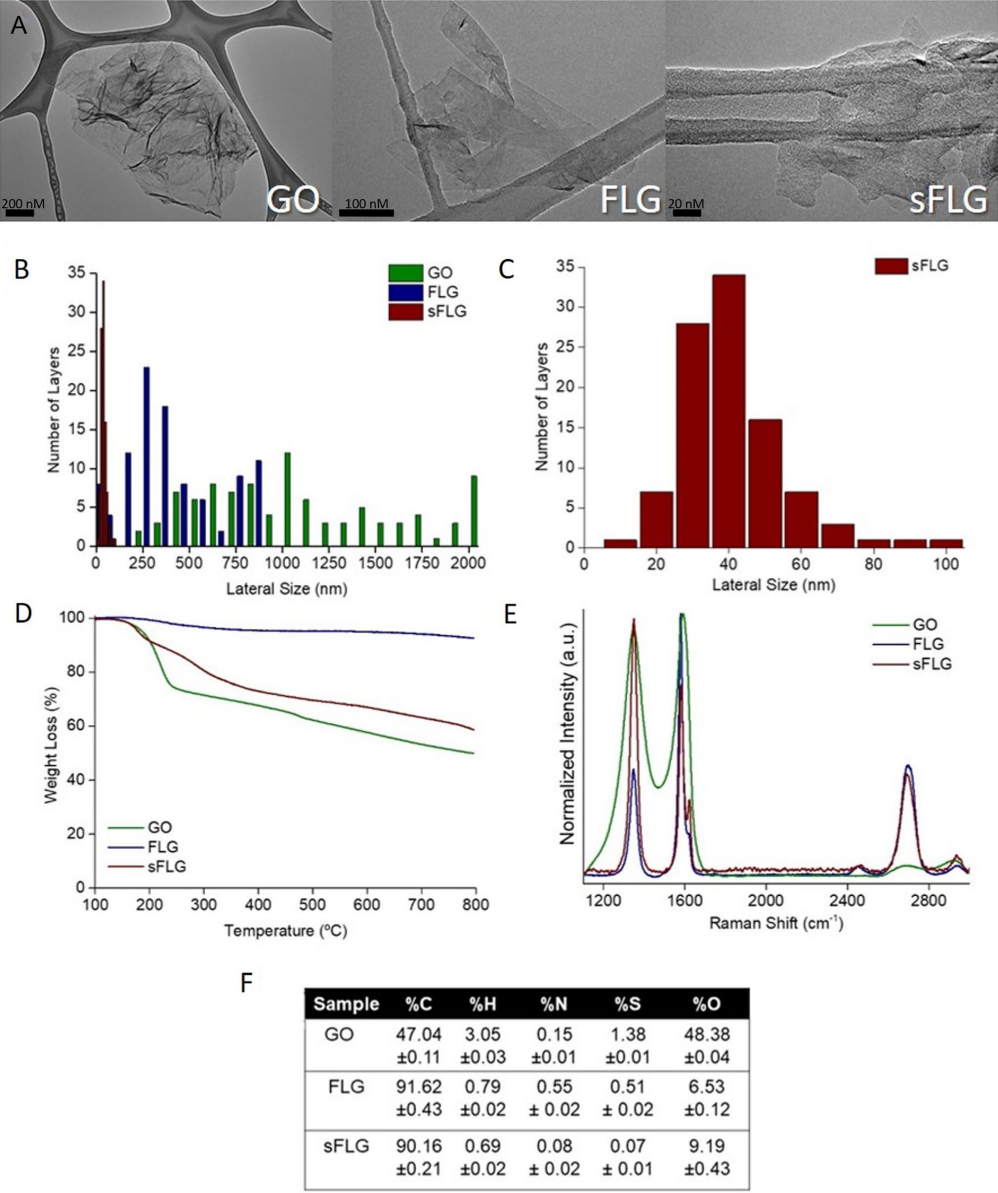
Characterization of GO, FLG, and sFLG: (**A**) HRTEM Image (GO scale bar: 200 nm; FLG scale bar: 100 nm; sFLG scale bar: 20 nm); (**B**) lateral size distribution of flakes; (**C**) lateral size distribution of sFLG; (**D**) TGA results in nitrogen atmosphere; (**E**) Raman spectra; and (**F**) elemental analysis of nanomaterials.

Figure 1A shows standard high-resolution transmission electron microscopy (HRTEM) images for GO, FLG, and sFLG. The size distribution of the graphene flakes shows completely different lateral sizes depending on the type of material (Fig. 1B and 1C), with an average length of 1.18 µm ± 994 nm for GO, 300 ± 23 nm for FLG, and 36.04 ± 15 nm for sFLG. Thermogravimetric analysis (TGA) (Fig. 1D) of GO, FLG, and sFLG was performed under a nitrogen atmosphere. The weight loss at a temperature of 600 °C—corresponding to the oxygen-containing groups on the graphene layers—was 57.30%, 4.81%, and 33.30% for GO, FLG, and sFLG, respectively. The significant mass loss of GO and sFLG between 100 and 300 °C was expected to decompose functional groups (–OH, –COOH, and –C–O–C)^30,31^ that are not found on FLG. Raman spectroscopy is illustrated in Fig. 1E, indicating the presence of the D band (1350 cm^–1^, related to some defects in the carbon rings), G band (1580 cm^–1^, associated to sp^2^ carbon bonds in the hexagonal structure), and 2D band (2700 cm^–1^, related to the number of graphene layers and the quality of carbon rings)^32^. For carbon nanomaterials, two main parameters need to be considered in Raman spectra: the intensity ratio between the D and G bands (I_D_/I_G,_), to quantify the density of defects in graphene^33^; and the shape of the 2D band, to determine the number of layers (N_G_)^34^. The I_D_/I_G_ values obtained for the nanomaterials were 0.94, 0.42, and 1.34 for GO, FLG, and sFLG, respectively. GO and sFLG showed the highest I_D_/I_G_ values due to these having a more significant amount of defects than FLG, which is consistent with the TGA results. The increased D band in sFLG is related to the small size of graphene layers compared to the number of functional groups at the edges. At the same time, GO shows a low intensity in the 2D band related to higher structural defects of its carbon rings^35^. In the case of FLG and sFLG, it was possible to calculate the average number of layers—three in each case^34^. Elemental analysis of GO, FLG, and sFLG (Fig. 1F) yielded a percentage of 48.37% oxygen in the GO sample, 6.53% in FLG, and 9.19% in sFLG—…results which are consistent with those obtained with other characterization techniques. The nanomaterial powders were re-dispersed in the different culture media (DMEM with/without FBS and completed BEGM) at 5 µg/ml (Supplementary Fig. 1A–C) and the colloidal stability of the nanomaterials was studied through UV–Vis absorption spectroscopy for 24 h, (see “Methods”). Supplementary Table 1 shows the average sedimentation at 2 h and 24 h for all the different nanomaterials. sFLG is the nanomaterial with the lowest sedimentation in all the different culture media after 24 h, which can give an idea about the delivered dose in each treatment. It is also important to note that although the sedimentation of GO after 2 h depends on the culture media, after 24 h there are no significant differences in the sedimentation of this nanomaterial in DMEM with FBS or in completed BEGM. Same results are observed for FLG and sFLG. These data can be explained due to the fact that BEGM incorporates complements that are similar to those found in FBS, such as different proteins or BPE (bovine pituitary extract).

### Graphene induces necrosis in primary human bronchial epithelial cells

GRMs can induce cell death by necrosis and apoptosis^36,37^. Necrosis is an uncontrolled mode of cell death involving loss of membrane integrity, which leads to activation of inflammation in vivo^38^. Previous works have shown that GRM-induced toxicity involves necrosis in different cell types and organs^28,37^, including lung tumor cells^39^. However, the toxicity of GRMs remains undetermined in normal, primary epithelial cells.

**Figure 2:**
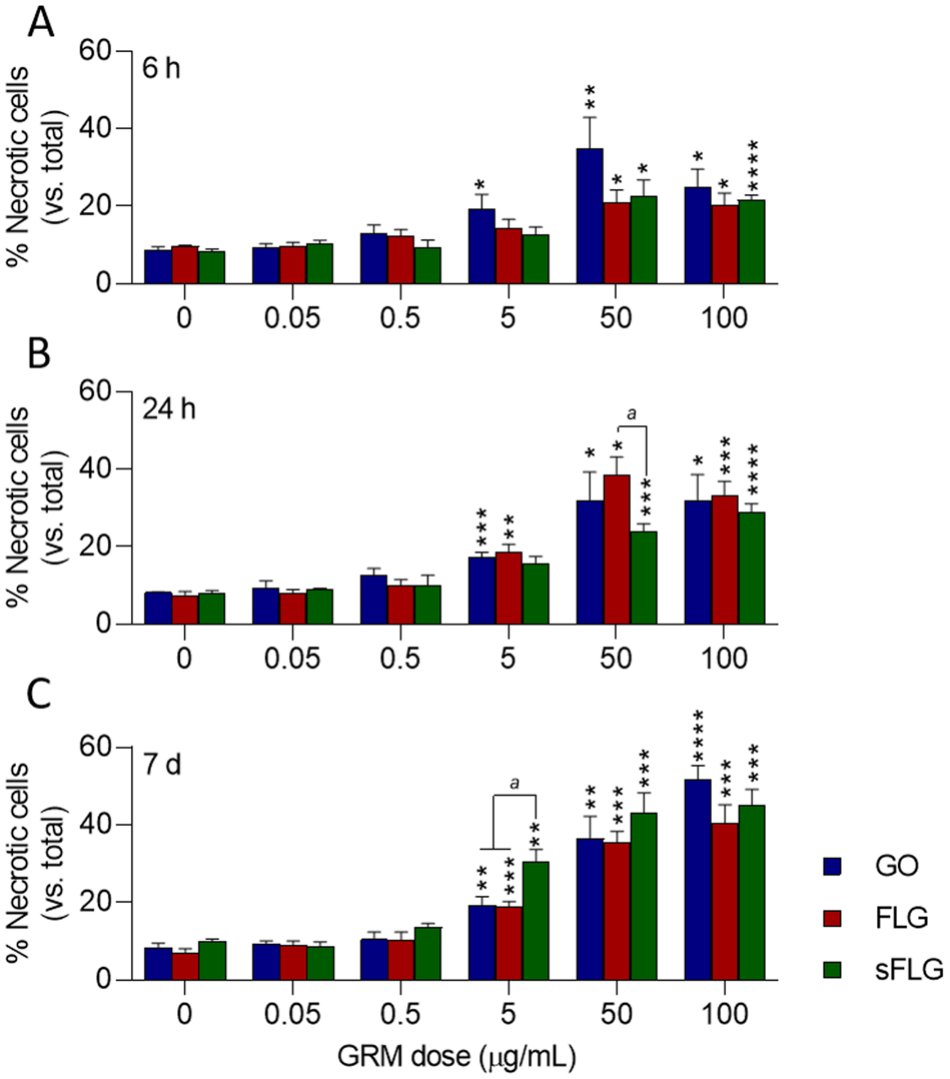
Effect of GO, FLG, and sFLG on NHBE cell necrosis: percentage of necrosis in NHBE cells treated with increasing concentrations of GO, FLG, or sFLG for 6 h (**A**), 24 h (**B**), and 7 days (**C**). Data are shown as percentage ± SEM (*p < 0.05; **p < 0.01, ***p < 0.001; ****p < 0.0001; *n* = 4).

In NHBE cells, low doses of the different GRMs did not increase necrosis after 6 h of exposure (Fig. 2A). A concentration of 5 µg/mL of GO—more oxidized—significantly increased necrosis (10.6%) compared to control (*p* < 0.05). Higher doses of GO, FLG, and sFLG (50 and 100 µg/mL) showed a significant and remarkable increase in necrosis, reaching more than 30% for 50 µg/mL GO (*p* < 0.01) (Fig. 2A). In cells exposed for 24 h, 5 µg/mL GO and FLG significantly increased necrosis to 17.3% (*p* < 0.001) and 18.7% (*p* < 0.01) (Fig. 2B). Higher doses of the different GRMs increased necrosis in a generalized way, reaching 38% for 50 µg/mL FLG (Fig. 2B).

When exposure to the different GRMs was extended up to seven days, necrosis drastically increased for all compounds. Compared to their respective controls, a 5 µg/mL dose of GO, FLG, and sFLG increased necrosis significantly. In particular, 5 µg/mL sFLG induced 27.5% of necrosis (Fig. 2C). Exposure to 100 µg/mL GO was the most harmful, damaging more than 50% of cells (Fig. 2C).

### Graphene induces apoptosis in primary human lung cells

Apoptosis is a type of programmed cell death essential for maintaining cell homeostasis. It is characterized by specific morphological nuclear changes such as condensation and fragmentation and the appearance of apoptotic bodies^36,40^. Apoptosis, as necrosis, is one of the main mechanisms of GRM-induced cell death^36^. Our results indicate a similar trend to that observed for necrosis, although percentages of apoptotic cells were consistently lower than necrotic ones (Supplementary Fig. 2).

In cells exposed for 6 h, a significant increase in apoptosis induced by 0.5 µg/mL FLG and sFLG was noted, reaching 7.7 and 6.8%, respectively (Supplementary Fig. 1). Although the effect did not seem to be dose-dependent, percentages increased to 10–12% for higher GRM concentrations (5–100 µg/mL). The same trend was observed at 24 h (Supplementary Fig. 2B) and seven-day exposures (Supplementary Fig. 2C)—the latter with apoptosis percentages above 20% at high concentrations (50–100 µg/mL). These results indicate that GRM-induced toxicity causes NHBE cells to die preferentially by physical damage rather than programmed cell death.

### Cytotoxic effect of graphene in A549 lung tumor cells

**Figure 3:**
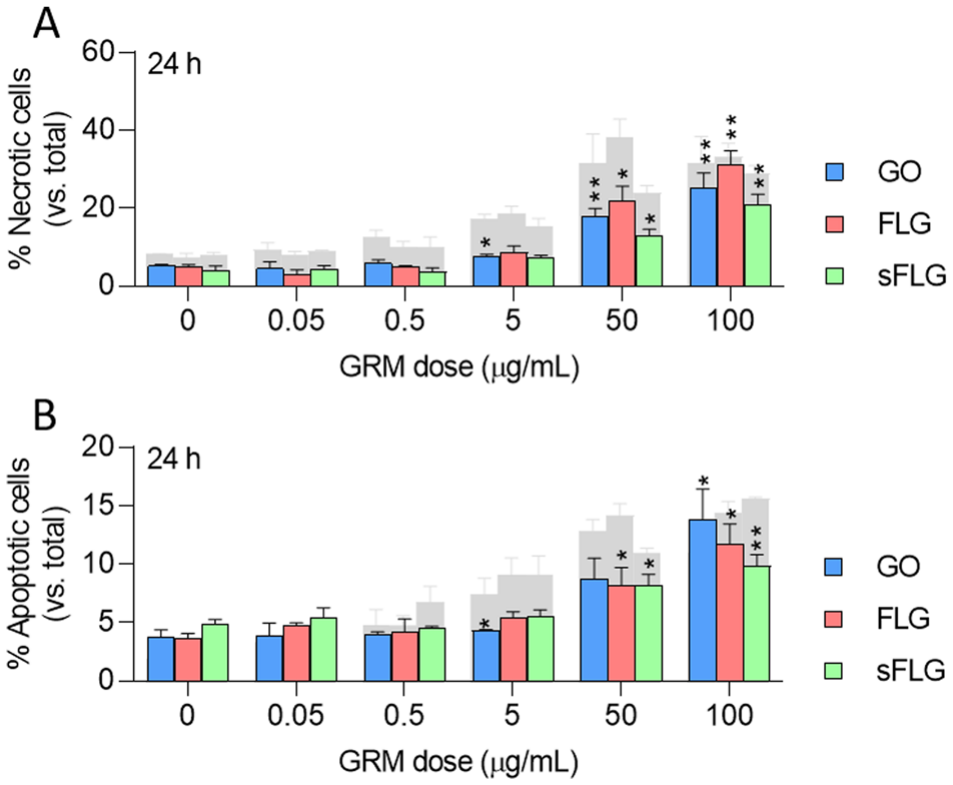
Effect of GO, FLG, and sFLG on A549 cell necrosis and apoptosis: percentage of necrotic (**A**) or apoptotic cells (**B**) in cells treated with GO, FLG, or sFLG for 24 h. Gray bars represent the levels of necrosis or apoptosis in NHBE. Data are shown as percentage ± SEM (*p < 0.05; **p < 0.01; *n* = 3).

A549 is the lung cell line most widely used to assess the toxicity of nanomaterials, including graphene^16,41,42^. First, we compared the morphological features between A549 and NHBE cells without observing differences in cell size and morphology (Supplementary Fig. 3A, B), indicating two phenotypically similar cell types. Then, we evaluated the toxicity of increasing doses of GO, FLG, and sFLG in A549 cells exposed for 24 h, comparing the results with those observed on NHBE cells (Fig. 3). GRMs induced a dose-dependent increase in necrosis, although the values were less than half those of NHBE cells (shaded bars) at doses between 0.05 and 5 µg/mL. This difference was reduced at higher concentrations (50–100 µg/mL) (Fig. 3A). A similar trend was observed in apoptosis, which was significant only for 50–100 µg/mL (Fig. 3B). A549 cells grow in a different culture medium than NHBE cells, which includes 10% fetal bovine serum (FBS). Previous research has shown that the presence of FBS in the medium can reduce graphene-induced cytotoxicity^43^. Therefore, to evaluate the possible effect of FBS, apoptosis and necrosis were assessed in A549 cells grown in the FBS-free medium for 6 and 24 h and exposed to 5 µg/mL GO, FLG, and sFLG for 24 h (Supplementary Fig. 4). No differences in the levels of necrosis (Supplementary Fig. 4A) and apoptosis (Supplementary Fig. 4B) were observed, suggesting that the presence of FBS was not critical for the cytotoxic effect of the different GRMs in A549 cells. Therefore, A549 cells appear to be more resistant than NHBE to the cytotoxic effects of GRMs and are insensitive at low concentrations—which would be a physiological dose in terms of possible inhalation.

### Graphene drastically reduces the viability of primary human lung cells

**Figure 4:**
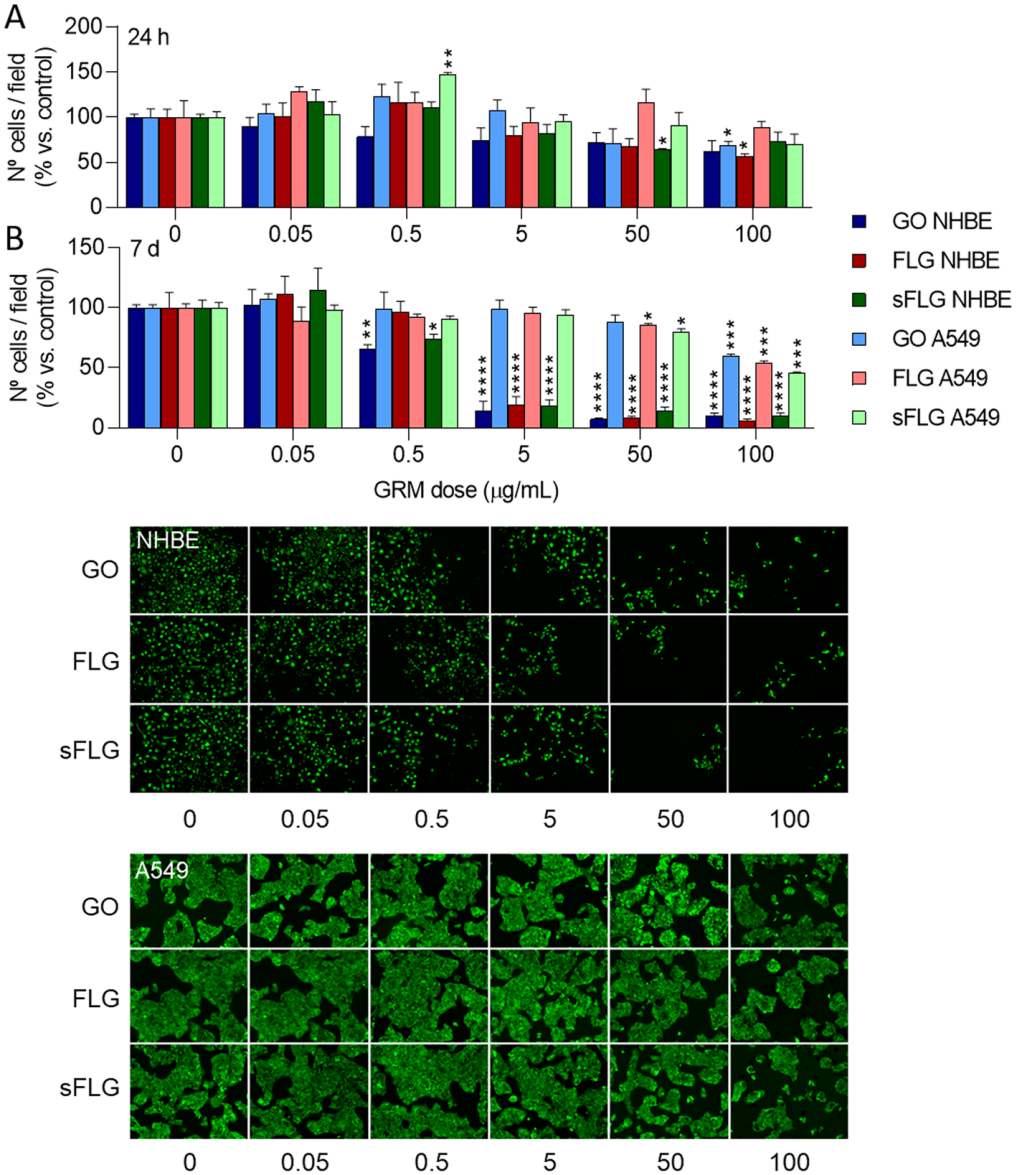
Effect of GO, FLG, and sFLG on cell viability. Percentage of viable NHBE and A549 cells treated with GO, FLG, or sFLG for 24 h (**A**) and 7 days (**B**). Data are shown as percentage ± SEM (*p < 0.05; **p < 0.01, ***p < 0.001; ****p < 0.0001; *n* = 3).

Prolonged exposure of NHBE cells to harmful compounds results in cell death and, consequently, the detachment of cells from the culture plate surface^44^. Our study used fluorescence microscopy to analyze the number of cells attached to the culture dish and the viability of the remaining cells. Short time exposure (6 h) of NHBE cells to GRMs alters the number of attached cells and their viability without reaching significance (Supplementary Fig. 5). Similarly, there is a dose-dependent trend to the number of cells per field decreasing in 24-h treatments, reaching significance at high concentrations—50 µg/mL sFLG and 100 µg/mL FLG generated reductions of 35.5% and 43.7%, respectively (Fig. 4A). There is no effect on A549 cells with GRM concentrations of 0.05–5 µg/mL (Fig. 4A). A reduction was detected for GO at 50 µg/mL and for all GRMs at 100 µg/mL, although always lesser than those values observed for NHBE cells (Fig. 4A). No differences were observed in the number of A549 cells cultured in medium with FBS and medium without FBS and exposed to 5 µg/mL of the different GRMs (Supplementary Fig. 4C). A seven-day exposure to GRMs profoundly impacted NHBE cell viability, significant for low doses of 0.5 µg/mL GO and sFLG. For doses of 5 µg/mL GO, FLG, and sFLG, there was a decrease of 86.2%, 81,1%, and 81,7%, respectively, which was even more significant for higher doses (50–100 µg/mL of GRMs reduced cell viability up to 90%) (Fig. 4B). Interestingly, for a seven-day exposure the effect on A549 cells was only observed at a concentration of 100 µg/mL (Fig. 4B).

### Graphene alters cytosolic and mitochondrial Ca^2+^ and reactive oxygen species in NHBE cells

**Figure 5:**
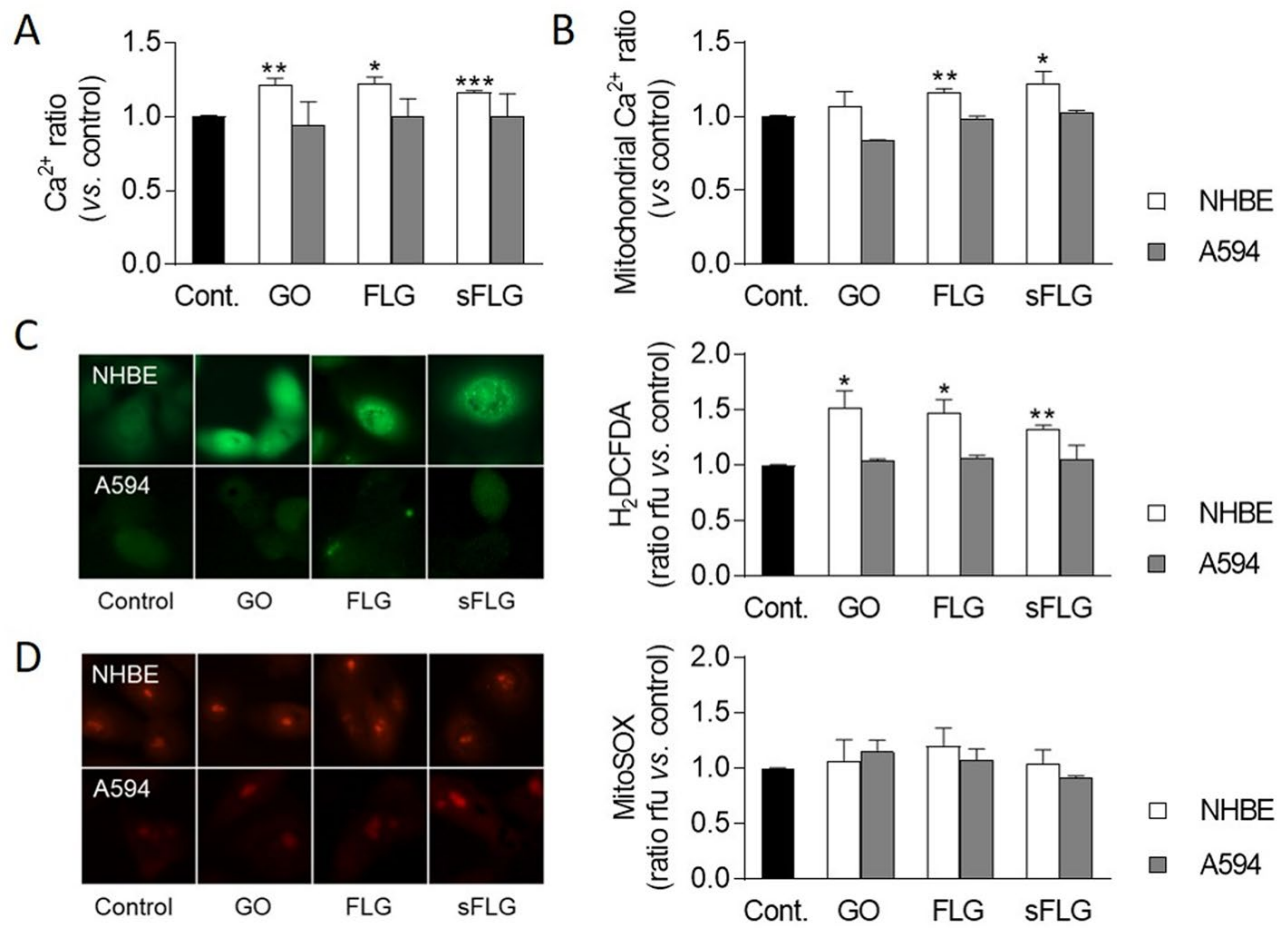
Effect of GO, FLG, and sFLG on Ca^2+^ homeostasis and ROS levels in NHBE and A549 cells: cytosolic (**A**), mitochondrial (**B**) Ca^2+^ ratio, H_2_O_2_ (**C**) and O_2_^–^ (**D**) in NHBE or A549 cells treated with 5 µg/mL GO, FLG, or sFLG for 24 h. Data are shown as mean ± SEM (*p < 0.05; **p < 0.01, ***p < 0.001; *n* = 3).

The next step was to examine the underlying mechanisms through which GRMs can induce cell death. Based on the results detailed above, experiments were performed in NHBE and A549 cells incubated for 24 h with a 5 µg/mL dose of GO, FLG, and sFLG. Cell morphology was determined as a standard measure of cell wellness status^45^. No morphological alterations in the width/length ratio were found (Supplementary Fig. 6A), although cell size decreased slightly in response to sFLG (Supplementary Fig. 6B). Calcium homeostasis and oxidative stress were then examined, as these are key processes related to graphene toxicity^3,28^. The free cytosolic Ca^2+^ level increased by 20% in NHBE cells treated with all GRMs but showed no change in lung tumor A549 cells (Fig. 5A). At the same time, there was a similar increase in mitochondrial Ca^2+^ for NHBE cells treated with FLG and sFLG—an effect not found in A549 cells (Fig. 5B).

One of the main mechanisms through which graphene generates toxicity is by increasing oxidative stress^46^. For that reason, hydrogen peroxide (H_2_O_2_) and superoxide anion (O_2_^–^) levels were analyzed. H_2_O_2_ and O_2_^–^ were determined by fluorescence microscopy in living cells with the H2DCFDA and MitoSOX probes. Levels of H_2_O_2_ increased by 51.3%, 46.3%, and 32.2% in NHBE cells treated with GO, FLG, and sFLG, respectively (Fig. 5C). No effect was observed in A549 lung tumor cells (Fig. 5C). On the other hand, O_2_^–^ levels were not altered by exposure to GRMs, neither on NHBE nor on A549 cells (Fig. 5D). Again, these results suggest that primary lung cells are more sensitive than the tumor cell line.

### Comparison of GRM-induced toxicity in NHBE cells with the effect of other toxic compounds

**Figure 6:**
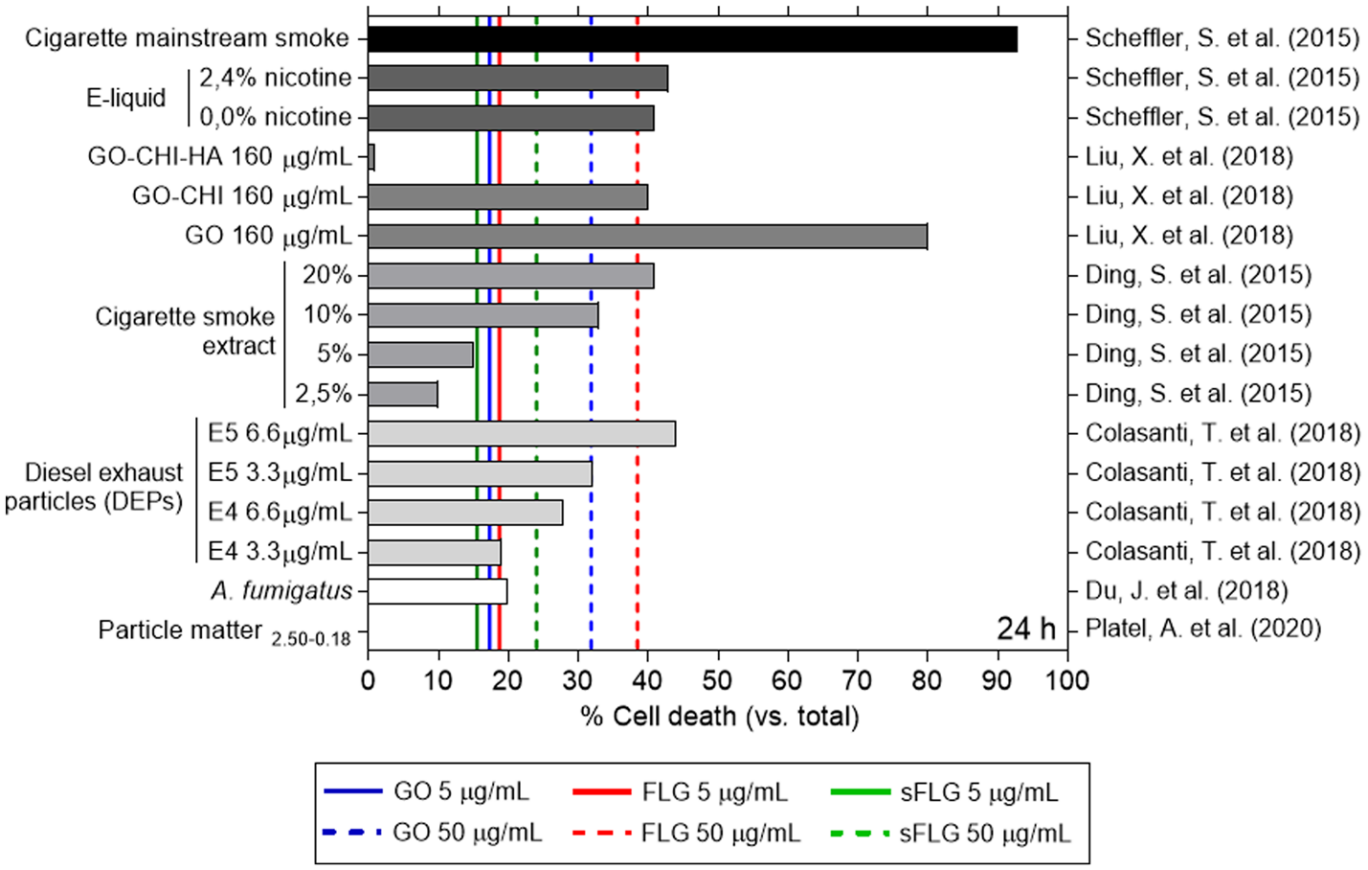
Effects of different compounds on NHBE cell death. The graph displays the cell death values of: cigarette mainstream smoke (CMS)^23^, E-liquid^23^, GO with chitosan (CHI) and hyaluronic acid (HA)^24^, cigarette smoke extract^25^, diesel exhaust particles (DEPs)^26^, *Aspergillus fumigatus*^27^, and particle matter (PM) (2.50–0.18 nm)^72^. Blue, red, or green lines represent the toxicity induced by GO, FLG, and sFLG.

NHBE cells have been used as a model in several in vitro lung toxicity studies^47–49^. Once it had been demonstrated that these cells were susceptible to GRM-induced cytotoxicity, our results were compared with existing data on the effect of other toxic compounds—i.e., cigarette smoke extract and diesel exhaust particles^25^, ^26^. After performing a database search, data on NHBE cell necrosis and apoptosis were compared to that extracted from research studies that used similar methodologies in terms of mode of exposure and incubation times. This comparison allowed us to establish that a 5 µg/mL dose of GO, FLG, and sFLG is as toxic as low concentrations of cigarette smoke extract^25^ or diesel exhaust particles^26^, whereas 50 µg/mL doses, especially in the case of FLG, damage cells in a similar magnitude to the highest doses of the compounds found in the literature^23,25,26^. Their toxicity was only exceeded by exposure to cigarette mainstream smoke^23^ (Fig. 6).

## Discussion

In recent years, many potential graphene applications have emerged across different research and innovation fields^3,7,8,50,51^. The growing interest in this material has led to an increase in its production—and, consequently, in human exposure to it. Many of these applications—e.g., face masks, sensors, and smart clothes—involve daily use and thus continuous exposure^52–54^. In order to create safe-by-design protocols, it is essential to study how graphene and GRMs interact with different human biological barriers, especially those that will come into direct contact with them^2,3^. Therefore, assessing how graphene interacts with the respiratory system, especially the interaction with the first chain of defense, the respiratory epithelium. These studies are crucial, for example, for setting occupational exposure limits. On the other hand, it is necessary to establish standardized criteria for this kind of studies^1,3^. The scientific community must conduct multiple studies, evaluating the potential impact of different GRMs at different doses and exposure times. In addition, it is necessary to define the most appropriate biological model to conduct these studies^3^. Finally, for an adequate toxicity assessment, different GRMs should be well-characterized through standardized protocols^55^.

The major potential routes of graphene into the body are inhalation, ingestion, and dermal adsorption^3^. Exposure to graphene is variable during its production process, involving direct interaction with the respiratory tract if adequate personal protective equipment is not used^56^. Concerns about the toxic effect of graphene on the lungs also extend to its integration into everyday products such as face masks^52^ and biomedical applications such as intranasal immunization^57^. Moreover, different studies on the biodistribution of graphene have demonstrated the presence of graphene in the lung after intravenous^58,59^, oral^60^, and intraperitoneal administration^61,62^. This suggests that the lung could also be damaged when other administration routes are used.

Different studies have evaluated the pulmonary toxicity of graphene in murine models in recent years, with contradictory results^3,63–68^. This is because the impact of graphene depends on its different physicochemical characteristics, concentration, and exposure time^3^. Bussy et al. recently observed GO inhalation could induce lung granulomas that persist up to 90 days after exposition^69^. This suggests that in vivo studies must evaluate its long-term effects. However, this is not very common. On the other hand, the in vivo studies published to date, evaluating different conditions and scenarios, required very large numbers of mice. To ensure the 3Rs principle and reduce costs and time, it is essential to refine the in vivo exposure conditions prior to conducting the experiments by using standardized in vitro toxicity assessment protocols. However, the choice of cellular models for in vitro study is a crucial issue that should not be taken lightly^70,71^.

In this work, we propose a model using primary normal human bronchial epithelial (NHBE) cells, which have been used previously to study particle-generated lung toxicity^23,25,26,72^. The gold standard to study graphene-induced lung toxicity is the lung tumor cell line A549^73,74^. Tumor cell models are cost-efficient, easy to use and provide an unlimited material supply. However, they do not have the same characteristics as normal cells, particularly regarding the composition and net charge of the plasma membrane or the oxidative stress response—all of which are critical for interacting with GRMs^3^. Indeed, some studies using A549 cells showed no toxicity after exposure to high doses (≥ 50 µg/mL) of graphene, indicating that this cell line is highly resistant to graphene-induced toxicity^75–77^.

Therefore, the use of the NHBE model offers a more realistic scenario for toxicity assessment. In this work, we have proposed a series of simple and reproducible toxicity determination procedures for identifying variations in cell viability, from slight to acute effects. The results indicate that low doses of different GRMs significantly increased NHBE cell death, an effect not observed in A549 cells (Figs. 2, 3, 4). Both cell lines, with similar morphological characteristics (Supplementary Fig. 3), showed different behaviour in response to GRMs. This effect could be enhanced by differences in the composition of the culture media of both models, especially by the presence of FBS in the culture medium of A549 cells, which may be associated with a higher protein corona in the graphene, therefore lower cytotoxicity^43^. Although the medium of NHBE cells lacks FBS, it incorporates high concentrations of different protein complements, producing the protein corona. However, to avoid this possible effect, the toxicity of the different GRMs (5 µg/mL) was studied in A549 cells grown in FBS starvation without observing significant differences. On the other hand, the results obtained in A549 cells were similar to those reported in previous works^46,65^. Differences were only due to the intrinsic characteristics of tumoral cells A549—i.e., membrane dynamics and resistance to oxidative stress^78^.

However, to avoid underestimating the real impact of GRM-based toxicity on lung cells and the cell model used, it is also crucial to combine different approaches. Studies published to date quantifying cytotoxicity by classical methods may underestimate the real in vitro cytotoxic impact of GRMs. Our study observed necrosis and apoptosis in cells exposed for seven days (Fig. 2C; Supplementary Fig. 2C) to 5, 50, and 100 µg/mL doses were much higher, since it was related to a very small proportion of surviving cells (Fig. 4). The substantial increase in cell death at seven-day exposures led us to focus our attention on a 24-h exposure time—which is also the standard exposure time in toxicity studies. Moreover, our study further evaluated other indirect parameters of cell damage, such as alteration in Ca^2+^ homeostasis and ROS levels. We observed that low doses of GRMs altered these parameters only in NHBE cells (Fig. 5).

Our study assessed the toxicity of three well-characterized GRMs with different lateral sizes and oxidation degrees. Regarding necrosis, 5 and 50 µg/mL GO (more oxidized) generated an immediate and acute increase in this parameter compared to FLG and sFLG, which was maintained over time (Supplementary Fig. 7). On the other hand, the size of the graphene was determinant in cytotoxicity at long times and low doses, as suggested by the high toxicity effect of seven-day sFLG exposure. This result could also be due to the fact that sFLG showed the lowest sedimentation after 24 h (Supplementary Fig. 1), which could imply a higher interaction with the cells. This difference was not observed at higher doses since the level of cytotoxicity generated was extremely high. This trend was not observed regarding apoptosis, highlighting the importance of combining different approaches to assess toxicity in the same study.

It has been fully demonstrated that small particles harm the lung^79^, and graphene is no exception. The toxicity of many of these particles has been studied previously using the NHBE cell line. Therefore, to put our results into context, we compared graphene-induced toxicity levels in NHBE cells with those of other toxic particles analyzed using the same cell model. The toxicity levels induced by 5 µg/mL doses of GO, FLG, and sFLG were comparable to those generated by low doses of toxic compounds such as DEPs^26^ and cigarette smoke extracts^25^. For 50 µg/mL doses (particularly FLG), toxicity levels were similar to those induced by high doses of DEP compounds or electronic cigarette smoke extracts^23,25,26^. For example, DEPs are generated by diesel engines, one of the most important sources of anthropogenic particulate matter emissions. These particles generate cytotoxicity in various cells, including NHBE^80–82^. Remarkably, different studies show that exposure to even low doses of these toxic compounds has a detrimental effect on human health^23,25,26,72^. The results obtained in our study allow us to conclude that, for NHBE cells, a 5 µg/mL dose of GRMs (considered as low) generated toxicity after 24 h of exposure, and a dose of 50 µg/mL was as toxic as higher doses of other, well-studied toxic nanoparticles.

## Conclusions

The management of graphene derivatives for their integration into everyday applications such as face masks can involve regular direct contact between the nanomaterials and the lung barrier. For this reason, it is essential to design accurate, fast, and easy-to-use screening protocols to (1) assay the toxicity of current and potential novel GRMs prior to their use in commercial applications, and (2) to increase the safety measures during their preparation and handling at research laboratories and companies. For the first time, the present work evaluates the harmful effect of different, well-characterized GRMs in a 2D model of primary human bronchial epithelial cells. This model allowed us to ascertain that the toxicity of several materials such as GO, FLG, and sFLG could be underestimated when using the current standard model, the lung tumor cell line A549. Indeed, our results indicated that lung cytotoxicity is proportional to the size and oxidation degree of the compound, with GO being the most toxic one tested—as lethal as cigarette compounds or DEPs even at low doses of 5 µg/mL. The use of primary, non-immortalized, and non-tumorigenic cells can provide a more accurate assessment of the interaction between GRMs and human lung cells—providing essential information for further testing in animal models, thus allowing the fulfillment of the Three R's principle.

## Methods

### GO synthesis

GO was kindly provided by Grupo Antolin (Burgos, Spain). Before its use, the material was washed to eliminate acid traces until the pH of the GO aqueous suspension was ∼5 in several cycles of Milli-Q water addition, re-dispersion, and centrifugation (4000 rpm, 30 min). The final suspension was lyophilized at a temperature of − 80 °C and pressure of 0.005 bar to obtain powdered GO.

### FLG and sFLG synthesis

FLG and sFLG were prepared by ball milling treatment using melamine^83^ and glucose^84^ as exfoliating agents, respectively, using a Retsch PM 100 planetary mill in both cases.

Briefly, for FLG, graphite (7.5 mg SP-1 graphite powder, purchased from Bay Carbon, Inc.) and melamine (22.5 mg, Sigma-Aldrich, ref. M2659) were mixed in a 25 mL stainless steel jar with ten stainless steel balls (1-cm diameter) and treated at 100 rpm for 30 min at room temperature and air atmosphere. After that, the resultant solid was dispersed in 20 mL of water for further dialysis at 70 °C, changing the washing water periodically (five changes every 120 min, including one overnight). Finally, the dispersion was left for five days to allow the sedimentation of graphite; the supernatant was extracted and lyophilized at a temperature of − 80 °C and pressure of 0.005 bar.

For sFLG, graphite (75 mg SP-1 graphite powder, purchased from Bay Carbon, Inc.) and D-glucose (4.5 g, purchased from Panreac) were mixed in a 250 mL stainless steel jar with 15 stainless steel balls (2-cm diameter). The jar was introduced in the planetary ball-milling machine at room temperature and air atmosphere for 4 h. The obtained solid was dispersed in 100 mL of water for further centrifugation (1500 rpm for 15 min) to remove non-exfoliated graphite and partial glucose. The supernatant was dialyzed at 70 °C to remove the glucose, changing the washing water periodically (seven changes every 90 min, including one overnight). The resulting dispersion was left to rest for five days at room temperature and air atmosphere. Then, the supernatant was lyophilized at a temperature of − 80 °C and a pressure of 0.005 bar. The colloidal stability in different culture media was studied using a UV–vis-NIR spectrophotometer (UV–Vis Cary 5000) with 1 cm quartz cuvettes^85^. The concentration of the nanomaterials was determined from the optical absorption at 386 nm for GO and at 660 nm for FLG and sFLG, during 24 h at different intervals, and using the calibration lines reported in Supplementary Table 2–4.

### Primary NHBE cells culture

Primary normal human bronchial epithelial (NHBE) cells were obtained from LONZA Walkersville Inc. (NHBE CC-2540; Lonza) from a single anonymous female donor, who was a non-smoker with no respiratory pathology. NHBE cells were seeded and grown according to the manufacturer's instructions. Briefly, cells were passaged once into a T25 flask in BEBM Bronchial Epithelial Cell Growth Basal Medium (CC-3171, Lonza) with BEGM Bronchial Epithelial Cell Growth Medium SingleQuots Supplements and Growth Factors, containing Bovine Pituitary Extract [BPE], Hydrocortisone, human Epidermal Growth Factor [hEGF], Epinephrine, Transferrin, Insulin, Retinoic Acid, Triiodothyronine, and Gentamicin/Amphotericin-B (CC-4175, Lonza). The growth media was changed every 48–72 h. When cells exceeded 45% confluence, the volume of the medium was doubled. Once cells reach 75–85% confluence, cells were re-seeded at 100,000 cells/T25 flask. Cells were passaged every seven days or when 85% confluency was reached. We used Clonetics ReagentPack (CC-5034, Lonza) for cell subculture with HEPES Buffered Saline Solution, Trypsin/EDTA, and Trypsin Neutralizing Solution. Cells were maintained at 37 °C in a 5% CO_2_ atmosphere. All experiments were performed between passages 1–5.

### Lung tumor A549 cell culture

Human lung cancer cell line A549 was purchased from ATCC (ATCC® CCL-185). A549 cells were seeded and grown according to the manufacturer's instructions. Briefly, cells were maintained in Dulbecco’s modified Eagle’s medium (DMEM) (#D6546; Sigma-Aldrich) with 10% fetal bovine serum (FBS) (#F4135; Sigma-Aldrich), 1% L-glutamine (#G7513; Sigma-Aldrich), and 1% Antibiotic Antimycotic Solution (#A5955-100ML Sigma-Aldrich) at 37 °C in a 5% CO_2_ atmosphere. Cell medium was renewed 2–3 times per week. The subcultivation ratio used was 1:8.

### Exposure of lung cells to GRMs

GO, FLG, or sFLG (0.05, 0.5, 5, 50, and 100 µL) were added to NHBE and A549 cells cultured in monolayers for up to 6 h, 24 h, and 7 days, depending on the assay. For seven-day incubation, cells received fresh medium at 72 h after GRM treatment.

### Determination of apoptosis and necrosis

Viability and necrosis were performed as reported in earlier studies^28,86,87^. Briefly, NHBE or A549 were seeded in 96-well cell culture plates and incubated for up to 6 h (10.000 cells/well), 24 h (10.000 cells/well), and 7 days (2.500 cells/well) with GO, FLG, or sFLG at increasing concentrations (0.05, 0.5, 5, 50, and 100 µg/mL). For FBS starvation tests, A549 cells were pre-cultured for 6 h or 24 h in a serum-free medium. Cells were then incubated with 10 μg/mL ethidium bromide (EtBr) (#46,067; Sigma-Aldrich) and 1 μM Calcein-AM (#C34852; Thermo Fisher). Viable cells, stained with green Calcein-AM, and necrotic cells, stained with red EtBr, were determined by fluorescence microscopy using a Cytation 5 Cell Imaging Multi-Mode Reader (20 × objective; BioTek) and analyzed with ImageJ 1.53. After image acquisition in living cells, samples were fixed and permeabilized in cold methanol for 4 min and then stained with 1 μg/mL Hoescht (#861,405; Sigma-Aldrich) to visualize DNA. Apoptosis was quantified by qualitative methods, as reported in earlier studies^28^. Results are presented as the number of cells per field or as a percentage of necrotic or apoptotic cells vs. total (*n* = 3).

### Morphological analysis

For morphological analysis A549 and NHBE cells were seeded in a 6-well plate (50.000 cells/well), after 24 h phase contrast images were acquired using an inverted microscope. Cell area, width and length were analysed using ImageJ (*N* > 50 cells).

### Determination of Ca^2+^ and mitochondrial Ca^2+^ in single cells

The intracellular Ca^2+^ levels were quantified using the probe Fluo-4 (#F23917; Thermo Fisher). Cells were seeded in 96-well plates (10.000 cells/well) and incubated for 24 h with 5 µg/mL of GO, FLG, or sFLG. Cells were then washed with PBS (5 min twice) and loaded for 30 min with 1 µM Fluo-4. After a brief washout, cells were imaged using a fluorescence microscope Nikon TiU (20 × objective) and analyzed using ImageJ 1.53. The results show the relative fluorescence units (RFUs) normalized vs. control levels (*n* = 3).

Levels of mitochondrial Ca^2+^ were quantified as described in earlier studies^86^. Briefly, cells were seeded in 96-well plates (10.000 cells/well) and incubated for 24 h with 5 µg/mL of GO, FLG, or sFLG. Cells were then loaded with 1 µM Calcein-AM (#C1430; Thermo Fisher). Cytosolic Ca^2+^ fluorescence (Calcein AM) was quenched with 1 mM CoCl_2_. After washing in fresh medium, images were acquired using a Cytation 5 Reader (Biotek) (20 × objective) and analyzed using ImageJ 1.53 (*n* = 3).

### Determination of O_2_^–^ and H_2_O_2_ in single cells

The level of intracellular reactive oxygen species was quantified in living cells using MitoSox (#M36008; Thermo Fisher) for O_2_^–^ and H_2_DCFDA (#C6827; Thermo Fisher) for H_2_O_2_. Cells were seeded in 96-well plates (10.000 cells/well) and incubated for 24 h with 5 µg/mL of GO, FLG, or sFLG. Cells were then washed with PBS (5 min twice) and loaded 30 min with 1 µM MitoSOX and 2.5 µM H_2_DCFDA. After 30 min, the excess dye was washed off with PBS (5 min once). For H_2_O_2_ quantification, cells were incubated at 37 °C DMEM in darkness for 30 min. Images were acquired using a Cytation 5 Reader (Biotek) (20 × objective) and analyzed using ImageJ 1.53. The results show relative fluorescence units (RFUs) normalized vs. control levels (*n* = 3).

### Statistics

Statistical analysis was performed with GraphPad Prism 8 (San Diego, CA, USA). To determine the statistical significance between control cells and GRM-treated cells we used Student t-test or one-way ANOVA (*p < 0.05; **p < 0.01, ***p < 0.001; ****p < 0.0001), followed by a Bonferroni's post-hoc test. All graphs were designed with GraphPad Prism 8 (San Diego, CA, USA). Dara are presented as mean ± standard error of the mean (SEM) of three independent experiments.

## Supplementary Information

Supplementary Information.

## Data Availability

The data sets used and/or analyzed during the current study are available from the corresponding author on request.
