# Supplementary material for: Rapid and efficient testing of the toxicity of graphene-related materials in primary human lung cells
Source: Sci Rep. 2022 May 10;12:7664. doi: 10.1038/s41598-022-11840-2 (PMC9088729; doi:10.1038/s41598-022-11840-2)
Supplement: Supplementary file 1 — Supplementary Information. [file 41598_2022_11840_MOESM1_ESM.docx]

**Rapid and efficient testing of the toxicity of graphene-related materials in primary human lung cells**

*Javier Frontiñan-Rubio^1,2^, Viviana Jehová González^2^, Ester Vázquez^2,3*^ and Mario Durán-Prado^1*^*

1 Universidad de Castilla-La Mancha. Faculty of Medicine, 13071, Ciudad Real, Spain

2 Universidad de Castilla-La Mancha. Instituto Regional de Investigación Científica Aplicada (IRICA), 13071, Ciudad Real, Spain

3 Universidad de Castilla-La Mancha. Faculty of Chemical Science and Technology, 13071, Ciudad Real, Spain

**Supplementary information**

**Supplementary information**

|  | **Sedimentation (%)** | | | | | |
| --- | --- | --- | --- | --- | --- | --- |
| **GRMs**  **Time** | **DMEM** | | **DMEM + 10% FBS** | | **Completed BEGM** | |
|  | **2h** | **24h** | **2h** | **24h** | **2h** | **24h** |
| **GO** | 5.6 | 26.0 | 3.4 | 16.2 | 17.4 | 18.6 |
| **FLG** | 6.9 | 9.8 | 5.6 | 19.6 | 4.1 | 20.5 |
| **sFLG** | 3.0 | 5.6 | 7.8 | 10.2 | 2.1 | 7.1 |

**Supplementary Table 1.** Percentage of sedimentation of GO, FLG, and sFLG in culture media during 24 h for DMEM with/without FBS and completed BEGM.

| **Samples** | **GO** | **FLG** | **sFLG** |
| --- | --- | --- | --- |
| **Intercept** | 0.34997 | 0.07229 | 0.05587 |
| **Slope** | 0.00617 | 0.00400 | 0.00206 |
| **R^2^** | 0.98412 | 0.99345 | 0.98539 |
| **Linear range (ug mL^-1^)** | 1.0-20.0 | | |

**Supplementary Table 2.** Analytical features for determining the concentration of GO, FLG, and sFLG in DMEM without serum.

| **Samples** | **GO** | **FLG** | **sFLG** |
| --- | --- | --- | --- |
| **Intercept** | 0.00222 | 0.0092 | 0.0012 |
| **Slope** | 0.00982 | 0.0043 | 0.0044 |
| **R^2^** | 0.99635 | 0.99477 | 0.99714 |
| **Linear range (ug mL^-1^)** | 0.5-5.0 | | |

**Supplementary Table 3.** Analytical features for determining the concentration of GO, FLG, and sFLG in DMEM with serum.

| **Samples** | **GO** | **FLG** | **sFLG** |
| --- | --- | --- | --- |
| **Intercept** | 0.01809 | -0.00374 | -6.316E-4 |
| **Slope** | 0.00159 | 0.00567 | 8.861E-4 |
| **R^2^** | 0.98674 | 0.99255 | 0.98742 |
| **Linear range (ug mL^-1^)** | 1.0-20.0 | | |

**Supplementary Table 4.** Analytical features for determining the concentration of GO, FLG, and sFLG in completed BEGM.

**
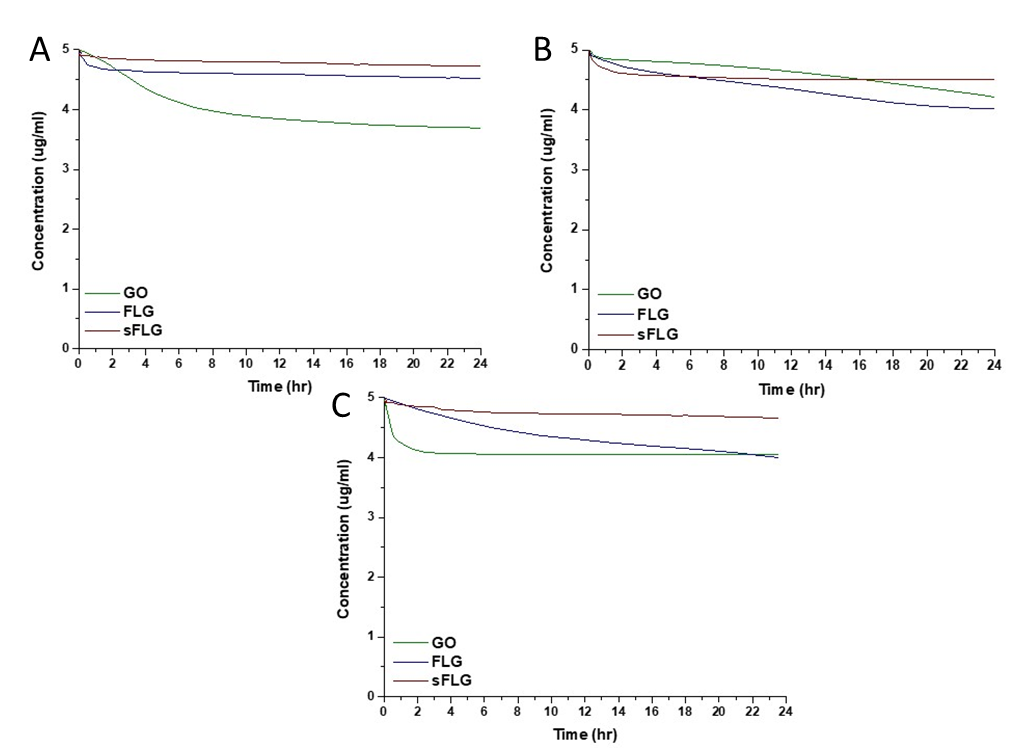
**

**Supplementary Figure 1.** Stability of GO, FLG, and sFLG in culture media during 24 h. (A) DMEM without FBS, (B) DMEM with 10% FBS, (C) completed BEGM.


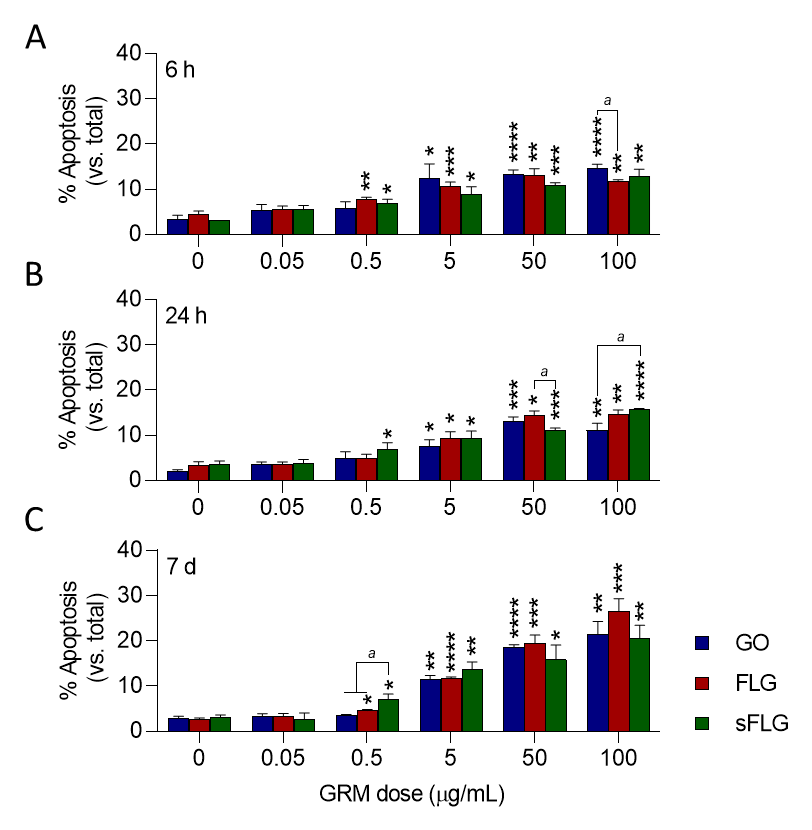


**Supplementary Figure 2.** Effect of GO, FLG, and sFLG on NHBE cell apoptosis: percentage of apoptosis in NHBE cells treated with increasing doses of GO, FLG, or sFLG for 6 hours (A), 24 hours (B), and 7 days (C) (*p<0.05; **p<0.01, ***p<0.001; ****p<0.0001 compared with the corresponding control group; a p<0.05 compared between different GBM; n=4).

*
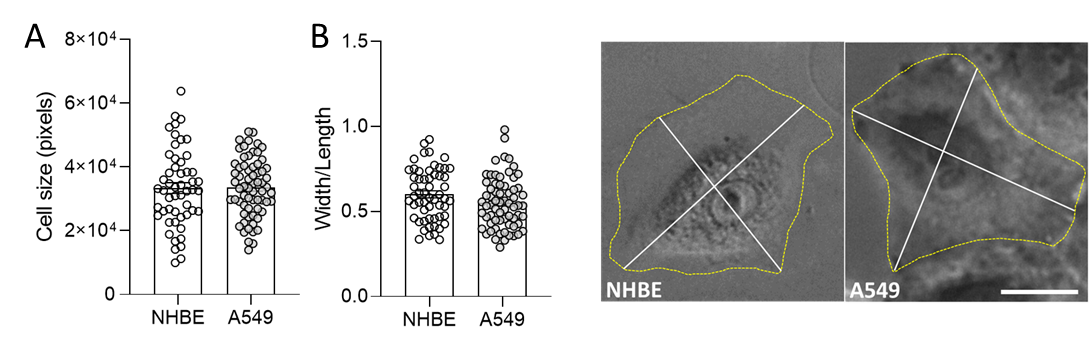
***Supplementary figure 3.** Morphological analysis of NHBE and A594 cells. Cell size (A) and width/length ratio (B). The image shows two representative cells with the cell area in yellow and the width and length in white. Scale bar: 20 µm. N>50 cells.


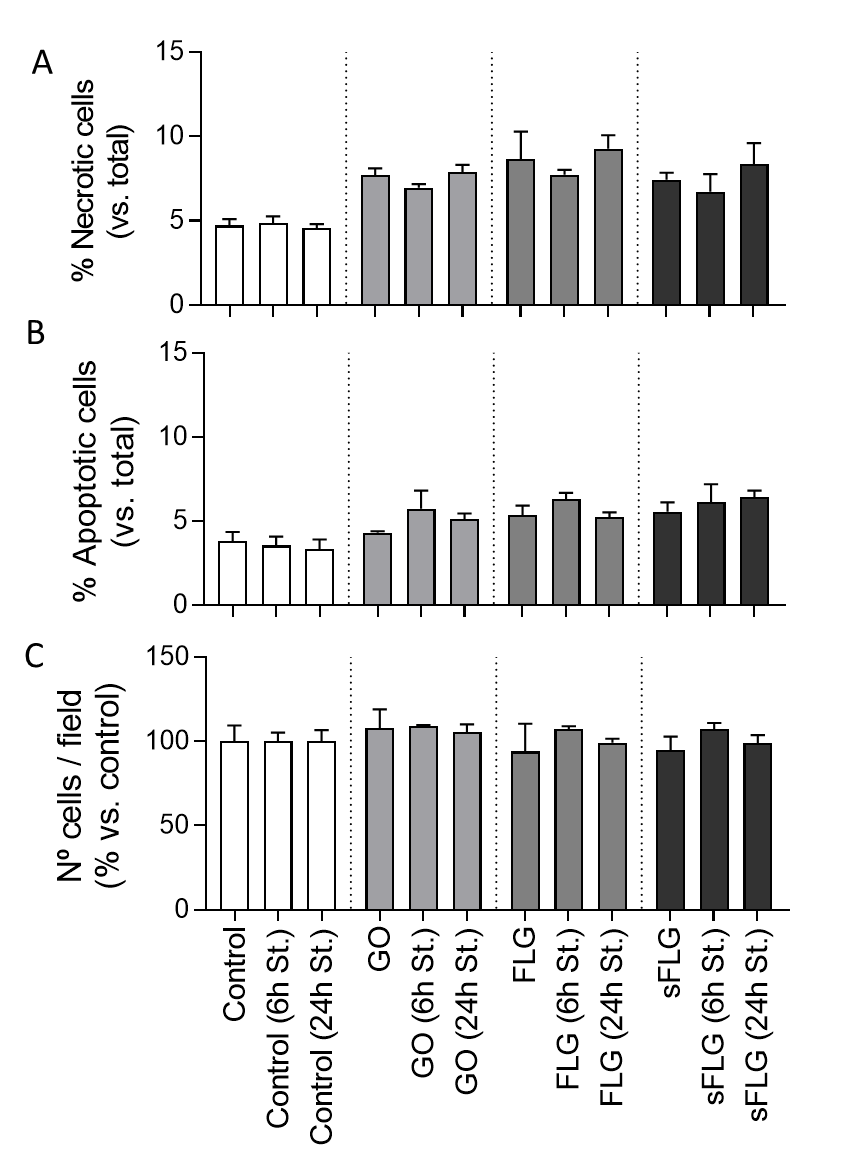


**Supplementary Figure 4.** Effect of 5 µg/mL GO, FLG, and sFLG on A549 cell damage. Percentage of necrosis (A), apoptosis (B) and total number of cells (C) in A549 cells cultured with 10% FBS and cells cultured in serum starvation conditions (6h or 24h starvation – St) treated with 5 µg/mL GO, FLG, or sFLG for 24h (n=3).

**Supplementary Figure 5.** Effect of GO, FLG, and sFLG on NHBE cell viability: number of viable cells treated with GO, FLG, or sFLG for 6 hours. Data shown as mean values ± SEM (n=3).


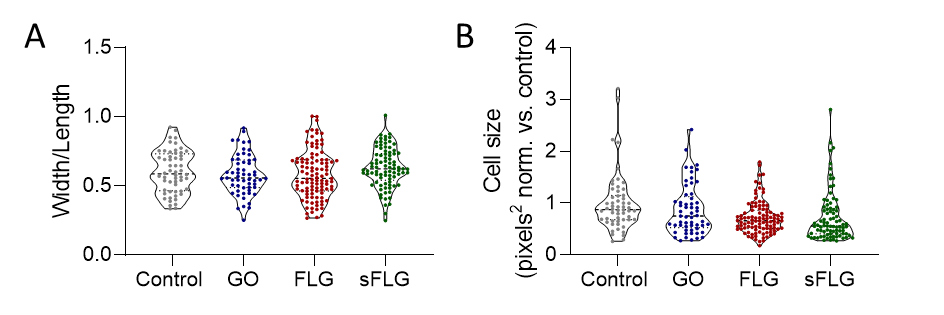


**Supplementary Figure 6.** Effect of GO, FLG, and sFLG on NHBE cell shape and size: cell shape measured as width/length ratio (A) and cell size (B) of cells treated with 5 µg/mL GO, FLG, or sFLG for 24 hours (>50 cells).

**Supplementary Figure 7.** Effect of GO, FLG, and sFLG on NHBE cell necrosis: percentage of necrosis in NHBE cells treated with 5 or 50 µg/mL of GO, FLG, or sFLG for 6 hours, 24 hours, and 7 days. Data shown as percentage ± SEM; n=4.
